# Peripheral Exudative Hemorrhagic Chorioretinopathy with and without treatment—Clinical and multimodal imaging characteristics and prognosis

**DOI:** 10.1371/journal.pone.0275163

**Published:** 2022-09-27

**Authors:** Margarita Safir, Ofira Zloto, Ido Didi Fabian, Iris Moroz, Dan D. Gaton, Vicktoria Vishnevskia-Dai

**Affiliations:** 1 Department of Ophthalmology, Shamir Medical Center, Zerifin, Israel; 2 Sackler School of Medicine, Tel-Aviv University, Tel Aviv-Yafo, Israel; 3 Ocular Oncology Service, The Goldschleger Eye Institute, Sheba Medical Center, Tel Hashomer, Herzliya, Israel; 4 Department of Ophthalmology, Rabin Medical Center, Petah-Tikva, Israel; Massachusetts Eye & Ear Infirmary, Harvard Medical School, UNITED STATES

## Abstract

**Purpose:**

To describe clinical and imaging characteristics of patients with Peripheral Exudative Hemorrhagic Chorioretinopathy (PEHCR), prognosis and treatment response.

**Methods:**

In this retrospective cohort study medical records of patients diagnosed with PEHCR in a tertiary medical center between 2008 and 2018 were reviewed. Collected data included demographics, medical history, ophthalmologic examination and multi-modal imaging including fundus autofluorescence, optical coherence tomography (OCT), ultrasound (US), fluorescein angiography and indocyanine green angiography when available. Bevacizumab treatment results were analyzed when applied.

**Results:**

35 eyes of 32 patients were included, with a female predominance (56.25%) and an average age of 79.0±9.87 years at presentation. Most common OCT and US findings were subretinal mass (68.75%), pigment epithelial detachment (30.00%) and atrophic changes (21.86%). Median follow-up period was 18.00 months (range 0–102). Visual acuity (VA) remained stable (39.29%) or improved (25.00%) in most cases available for follow-up. Treatment with intravitreal bevacizumab induced a statistically significant clinical resolution in 88.89% of eyes available for follow-up (8/9 eyes) (p = 0.02).

**Conclusions:**

PEHCR is presented with high clinical variability and generally good prognosis. This is the first publication demonstrating a statistically significant clinical resolution of disease following intravitreal bevacizumab injections.

## Introduction

Peripheral Exudative Hemorrhagic Chorioretinopathy (PEHCR) is a rare degenerative disorder, which affects mainly the peripheral retina. It may involve one or both eyes of the patient, and often mimics other, more ominous conditions.

This entity was first noticed by Reese and Jones in 1961 [1]. During the next two decades several authors described small series of similar patients [2–7]. In 1980, Annesley [8] used the term “peripheral exudative hemorrhagic chorioretinopathy” for the first time to describe peripheral chorioretinal lesions characterized by: subretinal pigment epithelial (RPE) or subretinal accumulation of either blood, exudates or both. These different types of lesions were present separately or simultaneously in a given patient. According to Annesley, PEHCR can be accompanied by additional abnormalities, including subretinal fluid, intraretinal blood, exudates or both in the overlying sensory retina, vitreous hemorrhage, and secondary alterations in the vascular pattern of the overlying retina. Annesley found, in concordance with further studies [8, 9], that PEHCR occurred most often in older Caucasian patients (mean age 69.9 years), with slight female predominance (55.5%).

Making the correct diagnosis in this population is challenging. Half of the patients in Annesley’s series were referred for examination due to suspected choroidal melanoma, vitreous hemorrhage with suspicion of underlying retinal detachment or break (14%), posterior vitreous detachment (4%), intraocular inflammatory process (4%), or retinal artery macroaneurysm (7%). The most important differential diagnosis to be made in this context is between benign PEHCR and ocular malignancies: while the hemorrhagic type may greatly resemble malignant melanoma, the exudative type often mimics primary vitreoretinal lymphoma (PVRL) [10–12]. In contrast to ocular malignancies, these lesions and patients have generally good prognosis [10], but long-term follow-up with various imaging modalities is often needed in order to distinguish between these entities [13]. A better understanding of typical disease presentation and course over time would contribute to our diagnostic abilities in such cases.

Several mechanisms have been proposed in the past for the pathogenesis of PEHCR. Subretinal neovascularization has been a leading theory [4, 6]. Although this hypothesis was supported by fluorescein angiography (FA) and indocyanine green angiography (ICGA) in several studies, no histologic evidence of such neovascularization could be found [5–7].

Later, optical coherence tomography (OCT) revealed a dome-shaped elevation of the pigment epithelium over vascular polyp-like structures, along with peripheral choriocapillary closure and dilated shunting vessels in affected eyes [6]. Therefore, several authors have recently proposed polypoidal choroidal vasculopathy (PCV) as the cause for PEHCR [5, 7, 10]. Although some patients did exhibit FA and/or ICGA findings consistent with peripheral PCV in the aforementioned studies, the authors admitted that these finding were evident only in a subgroup of patients. Nevertheless, based on this assumed pathophysiology, several attempts at PEHCR treatment with intravitreal anti-vascular endothelial growth factor (anti-VEGF) injections have been made [7, 9, 14–17]. All reports thus far have shown favorable, but statistically insignificant results, presumably due to small sample size and a relatively short follow-up time. Reviewing the literature up to the present, only 435 eyes with this rare disorder have been described. Each article addresses different characteristics of PEHCR, with few reports of long-term patient follow-up all the way from diagnosis, through management and to long term prognosis. The vast majority of these reports depict only short follow-up periods of up to 15 months [8, 10].

The purpose of our study is to present a thorough description of 35 eyes of 32 patients with PEHCR, diagnosed and treated in a single tertiary medical center and the outcome of a long follow-up period when available.

## Methods

This retrospective interventional cohort study was approved by the Institutional Review Board of our medical center and adhered to the tenets of the Declaration of Helsinki. Due to the retrospective nature of this study, the need for patients’ informed consent was waived by the ethics committee.

### Patients and data gathering

We reviewed the medical files of all patients with the diagnosis of PEHCR in Sheba Medical Center from 2008 until 2019. Materials included clinical examinations and various imaging findings including OCT, ultrasound (US), fluorescein angiography (FA) fundus photography, fundus autofluorescence (FAF) and ICGA when available. Standard conventional Heidelberg OCT was routinely performed for the macula, while OCT imaging of the PEHCR lesion itself was technically available only when the lesion approached the arcades or involved the macula. Treatment protocols when applied were recorded as well. All imaging studies were examined and last images were compared to the images at presentation. OCT angiography technology was not available at presentation. All US examinations were performed by the same experienced ophthalmologist, who also reviewed all OCT, FA and ICGA examinations.

One of two ocular oncology specialists examined all patients throughout their follow-up, and fundus color images were available for comparison of disease status. Ophthalmic examination included Snellen visual acuity (VA) charts converted to log minimum angle of resolution (Log MAR) values at the beginning and end of follow-up, intraocular pressure measurements with Goldman tonometer, slit lamp examination and dilated fundus examination.

Inclusion criteria were presence of at least one of the typical peripheral chorioretinal changes: peripheral chorioretinal lesions, sub retinal pigment epithelial (RPE) changes, accumulation of either exudates, sub retinal blood or both, pre retinal bleeding, or less commonly- sub retinal neovascularization. Patients in whom other diagnoses could not be ruled out at the time of data gathering were excluded from the study.

Treatment with intravitreal Bevacizumab was applied when lesions either involved or threatened the macula, were very large, induced vitreous hemorrhage, or when visual acuity was severely decreased in the fellow eye.

For the purpose of analysis clinical progression, stability and resolution were recorded according to the treating ophthalmologist’s description in last follow-up available for each patient. Disease resolution was defined using clinical and imaging findings, according to disease subtype: resolution of hemorrhage, leakage, sub retinal fluid and/or flattening of PED. Stable disease was defined as no change in the clinical or imagine presentation over time. Disease progression was defined as new clinical or imaging hemorrhage, leakage, sub retinal fluid or PED.

### Statistical analysis

Chi-square analysis was used to establish an association between dichotomous qualitative variables.

ANOVA was used to calculate difference in parametric variables between these groups. Independent and dependent t-tests were used in order to compare between parametric variables in different subtypes and at different timepoints respectively. For groups less than 30 patients, data were analyzed using non-parametric analysis tests. The overall significance level was set to an alpha of 0.05.

Statistical analysis was carried out using Microsoft Excel 16.1.1 (Microsoft Corporation, Redmond, WA, USA) and SPSS software version 23.0 (SPSS, Inc., Chicago, IL, USA).

## Results

### Demographic characteristics at presentation

Thirty-five eyes of 32 patients were diagnosed with PEHCR, of which 15/32 (46.88%) patients were male. Mean age was 79±9.88 years (range 60–94, median 80).

Frequently encountered general medical conditions included hypertension (24/32,75.00%), diabetes mellitus (14/32,43.75%), dyslipidemia (12/32,37.50%) and a positive history for malignancy in the past (10/32,31.25%).

At presentation 20/35 (57.14%) eyes were pseudophakic, 7/35 (20.00%) eyes had a concurrent diagnosis of non-exudative age-related macular degeneration (AMD), and 3/35 (8.57%) eyes had received intravitreal anti-VEGF injections prior to presentation for other indications.

### Clinical characteristics at presentation

Main clinical characteristics at presentation are described in **Table 1**. Three out of 32 patients had bilateral involvement (cases 7&8, 11&12, 17&18), not necessarily at presentation. Overall, 36.40% of eyes were asymptomatic, referred for investigation of an incidental retinal finding. The most common referral diagnosis was a choroidal mass raising suspicion of malignancy, most often choroidal melanoma (9/35 eyes, 25.7%) (**Table 2**). Lymphoma was the referral diagnosis in 2/35 eyes (5.71%), all of which had the exudative subtype.

**Table 1:** Demographic and clinical characteristics at presentation.

Case	Age	Gender	Side	Clinical subtype	Visual complaint	VA	Drusen	Pigment changes	RPE changes	Serous elevation	RD	PED	Macula involved	Num of foci	Num of lesions	Location					
																ST	IT	SN	IN	T	N
1	88	F	RE	Exudative	X	0.5				X				2	multiple					X	
2	80	F	RE	Combined	X	0.5								1	multiple		X		X		
3	83	F	LE	Exudative	X	2.3	X						X	2	multiple	X					X
4	79	M	RE	Exudative	X	1.4				X				1	multiple	X					
5	72	F	RE	Combined	X	0.5	X		X					2	multiple					X	
6	66	F	RE	Hemorrhagic		0.3		X				X		2	multiple					X	
7	83	F	RE	Combined		1.3					P			1	multiple	X					
8	85	F	LE	Hemorrhagic	X	0.7								1	single					X	
9	67	F	LE	Exudative		0.1	X	X	X					1	multiple					X	
10	77	M	RE	Combined		0.3			X					1	single		X				
11	60	F	RE	Exudative	X	0.2	X					X	X	1	multiple					X	X
12	60	F	LE	Combined	X	NLP	X				P	X	X	1	multiple					X	X
13	65	F	RE	Combined	X	0.7				X				2	multiple				X	X	
14	79	F	LE	Exudative		0.7			X					1	single				X		
15	70	M	LE	Exudative	X	2		X		X				1	multiple					X	
16	63	M	LE	Hemorrhagic		0.3	X	X	X			X		1	single		X				
17	94	M	LE	Hemorrhagic	X	0.3			X					1	single	X					
18	94	M	RE	Exudative		0.1	X		X					1	single	X					
19	87	M	LE	Hemorrhagic		1			X					2	multiple					X	X
20	85	M	LE	Hemorrhagic		0.5	X	X						1	single					X	
21	90	F	RE	Hemorrhagic		0			X			X		2	single					X	
22	84	F	RE	Combined	X	0.3			X	X				1	single	X					
23	75	F	LE	Hemorrhagic		0.3	X			X				1	multiple		X			X	
24	90	F	RE	Combined		0.4		X						1	multiple					X	
25	70	M	RE	Exudative	X	2.3		X			C			1	single		X	X			
26	88	F	RE	Combined		0.7		X	X					1	single	X	X				
27	88	F	RE	Exudative	X	2.3	X	X		X				1	single					X	
28	76	M	LE	Hemorrhagic		0.7		X	X					1	single			X	X		
29	76	F	LE	Combined	X	0.4	X	X						2	multiple				X		X
30	79	M	RE	Hemorrhagic		1		X	X					1	single		X	X			
31	65	M	RE	Exudative	X	0		X	X					1	multiple	X					
32	86	M	LE	Hemorrhagic	X	LP								1	multiple	X	X			X	
33	90	M	LE	Hemorrhagic	X	0.4								1	single		X		X	X	
34	85	F	RE	Exudative		0.8								1	single	X					
35	86	M	RE	Hemorrhagic	X	2.3								1	single					X	

Num- number; M- male; F- female; RE- right eye; LE- left eye; VA- visual acuity in logMAR; LP- light perception; NLP- no light perception; RPE- retinal pigment epithelium; RD- retinal detachment; PED- pigment epithelium detachment; ST- superotemporal; IT- inferotemporal; SN- superonasal; IN- inferonasal; T- temporal; N- nasal; P- partial; C-complete.

**Table 2:** Referral diagnoses before PEHCR diagnosis.

Referral Diagnosis	N-Eyes
Unspecified choroidal mass	18/35 (51.43)
Melanoma	9/35 (25.71)
Metastasis	3/35 (8.57)
Lymphoma	2/35 (5.71)
Paraneoplastic syndrome	2/35 (5.71)
Hemangioma	1/35 (2.86)

N = number of eyes. In parenthesis- % of eyes in the group.

PEHCR- Peripheral Exudative Hemorrhagic Chorioretinopathy.

Two eyes presented with severely decreased vision (LP and NLP), excluding them from the calculation- the mean visual acuity (VA) at presentation was 0.72±0.73 logMAR (range 0–2.3, median 0.4). Apart from 3 cases of macular involvement, VA compromise was caused by disc atrophy (1 case), AMD (4 cases), cataract (1 case) total retinal detachment (RD) at presentation (1 case) or previous macula-involving RD (1 case).

**Fig 1:**
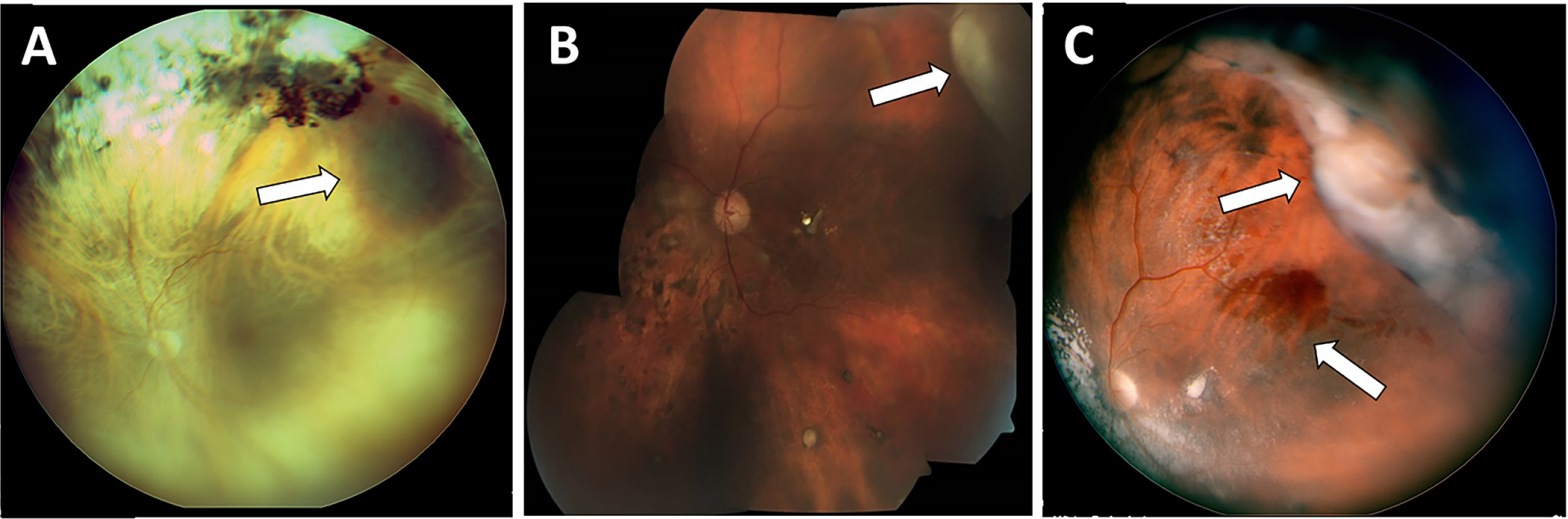
Clinical subtypes of PEHCR. Three different subtypes were observed in our cohort (arrows): **A**, Hemorrhagic; **B**, Exudative; **C** combined.

Three clinical subtypes were noted: exudative 12/35 eyes, (34.29%), hemorrhagic 13/35 eyes (37.14%) and combined 10/35 eyes (28.57%) (**Fig 1**, **Table 3**). In patients with bilateral disease a different subtype could be found in each eye.

**Table 3:** PEHCR clinical subtypes.

Clinical and imaging parameters		Combined	Exudative	Hemorrhagic	P value
**At presentation (35 eyes, 32 patients)**					
Age		77.5 ± 9.66	77.33 ± 10.74	78.77 ± 10.28	0.477
VA		0.57 ± 0.31	1.06 ± 0.94	0.65 ± 0.60	0.218
BCVA		0.52 ±0.33	1.01 ± 0.97	0.59 ± 0.62	0.237
**Location of lesions**					
	Temporal	4/10 (40%)	5/12 (41.67%)	9/13 (69.23%)	0.286
	Inferotemporal	3/10 (30%)	1/12 (8.33%)	5/13 (38.46%)	0.006
	Superotemporal	3/10 (30%)	5/12 (41.67%)	2/13 (15.38%)	0.345
	Nasal	2/10 (20%)	2/12 (16.66%)	1/13 (7.69%)	0.676
	Inferonasal	3/10 (30%)	1/12 (8.33%)	2/13 (15.38%)	0.397
	Superonasal	0/10 (10%)	1/12 (8.33%)	2/13 (15.38%)	0.588
	Any temporal lesion	9/10 (90%)	11/12 (91.67%)	12/13 (92.31%)	0.980
	Any nasal lesion	4/10 (40%)	4/12 (33.33%)	4/13 (30.77%)	0.895
Single focus		7/10 (70%)	10/12 (83.33%)	10/13 (76.92%)	0.759
Single lesion		3/10 (30%)	5/12 (41.67%)	9/13 (69.23%)	0.147
US base mm		9.34 ± 2.47	7.13 ± 3.97	8.82 ± 5.40	0.665
US thickness mm		2.15 ± 1.25	2.82 ± 1.48	2.39 ± 1.19	0.603
**On follow-up (28 eyes, 25 patients)**					
Age		76.11 ± 9.13	78.33 ±10.84	80.9 ± 10.06	-
VA on follow-up		0.85 ± 0.70	1.13 ± 0.73	0.42 ± 0.24	0.044
BCVA on follow-up		0.83 ± 0.71	1.13 ± 0.73	0.63 ± 0.07	0.032
US base mm		-	6.27 ± 3.98	7.98 ± 3.02	0.257
US thickness mm		-	2.14 ± 1.15	1.56 ± 0.73	0.190
**Disease course**					
	Resolution	4/9 (44.44%)	3/9 (33.33%)	7/10 (70%)	-
	Stable	3/9 (33.33%)	3/9 (33.33%)	3/10 (30%)	
	Progression to resolution	-	2/9 (22.22%)	-	
	Progression	2/9 (22.22%)	1/9 (11.11%)	-	
**Change of subtype**					
	Combined	-	3/9 (33.33%)	2/10 (20%)	-
	Exudative	3/9 (33.33%)	-	-	
	Hemorrhagic	1/9 (11.11%)	-	-	
				

N = number of eyes. In parenthesis—% of eyes in the group. Calculated values represent the average and standard deviation.

PEHCR- Peripheral Exudative Hemorrhagic Chorioretinopathy; VA- visual acuity; BCVA- best corrected visual acuity; US- ultrasound. mm = millimeter

One patient in the hemorrhagic group had LP visual acuity at presentation, he was not available for follow-up. One patient in the combined group had NLP visual acuity at presentation and on follow-up.

The vast majority of eyes had only peripheral involvement, with only 3/35 eyes (8.57%) exhibiting macula involving disease. Signs of concomitant macular AMD including drusen and/or retinal pigment epithelium (RPE) changes were seen in 21/35 of affected eyes (60.00%), even without an official diagnosis of AMD.

At presentation 27/35 (77.14%) eyes had a single focus of disease (defined as the location of a single lesion, or a concentration of several adjacent lesions) with a varying number of lesions within the involved focus. A lesion was defined as a single area affected with the disease ranging from sub retinal pigment epithelial (RPE) changes, sub retinal accumulation of either blood, exudates or both, sub retinal neovascularization, pre and sub retinal bleeding.

The most common locations of lesions at presentation were extramacular: temporal in 14/35 (40.90%) eyes, superotemporal in 10/35 (28.57%) eyes, and inferotemporal in 9/35 (25.71%) eyes. Altogether, 29/35 eyes (82.86%) of eyes had at least one lesion in the extramacular temporal half, whereas only 11/35 (31.80%) eyes had a lesion in the extramacular nasal half.

### Imaging findings

**Fig 2:**
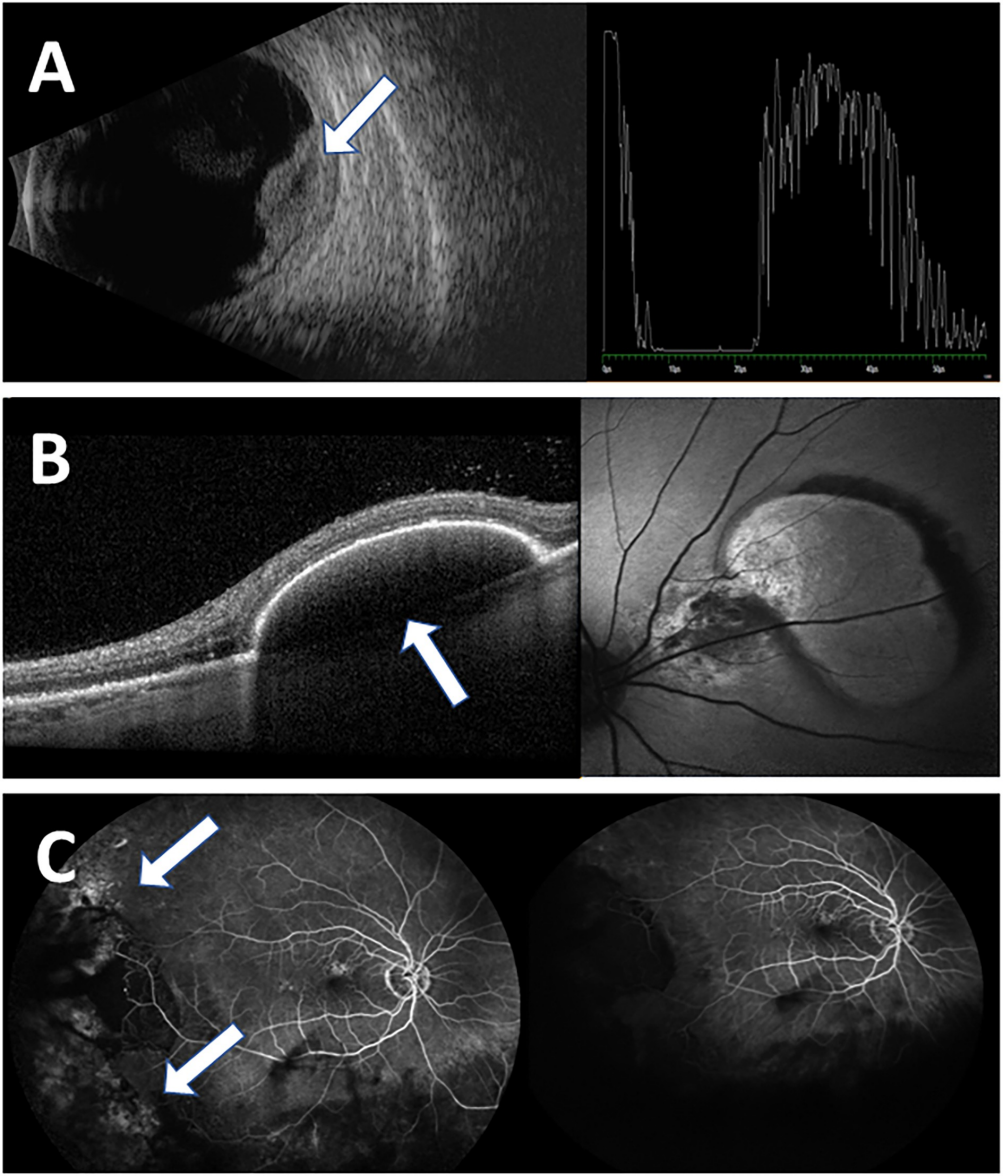
Main imaging findings of PEHCR in our cohort. The imaging modalities used for evaluation and follow-up of our patients included mostly ultrasound (US) and optical coherence test (OCT), in addition fluorescein angiography (FA) and Indocyanine Green Angiography (ICGA) in several patients. **A**, US image, depicting a choroidal mass (B-scan right side) with a high internal reflectivity (A-mode left side, arrow); **B**, The most common finding at presentation on OCT was PED (30%) (arrow) **C**, FA imaging reveals mostly hyperflourescent lesions (arrows) (55.56%), with no evidence of CNV.

The imaging modalities used for evaluation and follow-up of our patients included mostly US A mode and B scans (26/35 eyes), and when applicable- optical coherence test (OCT) (20/35 eyes). Fundus photography, fundus autofluorescence (FAF), fluorescein angiography (FA) and indocyanine green angiography (ICGA) were performed in the more recent cases, when this technology became widely available (**Fig 2**). **Table 4** summarizes main imaging findings.

**Table 4:** Main imaging findings.

Modality and findings			At presentation	Last follow-up visit	P value
**US**					
	Average base mm (n)		8.58±4.00 (20)	7.34±3.03 (8)	0.66
	Average thickness mm (n)		2.46±1.25 (26)	1.82±0.87 (9)	0.14
	Internal reflectivity:				
		Low	3/26 (11.53)	1/8 (12.50)	0.33
		Moderate	9/26 (32.62)	5/8 (62.50)	
		High	14/26 (53.85)	2/8 (25.00)	
	PVD		15/32 (46.86)	7/11 (63.64)	0.34
	Dome shaped mass		14/32 (43.75)	4/11 (36.36)	0.67
	Mass		10/32 (31.25)	0/11	0.04
	Choroidal thickening		7/32 (21.86)	2/11 (18.18)	1.00
	Retinal detachment		7/32 (21.86)	2/11 (18.18)	1.00
	Vitreous opacity		3/32 (9.38)	0/11	0.56
	Schisis		2/32 (6.25)	2/11 (18.18)	0.27
**OCT**					
	PED		6/20 (30.00)	1/10 (10.00)	0.37
	Macular RPE thickening/irregularity		6/20 (30.00)	2/10 (20.00)	0.68
	Scaring		4/20 (20.00)	4/10 (40.00)	0.38
	Subretinal fluid		4/20 (20.00)	1/10 (10.00)	0.64
	Choroidal elevation		3/20 (15.00)	2/10 (20.00)	1.00
	Subretinal deposits		3/20 (15.00)	3/10 (30.00)	0.37
	ERM		2/20 (10.00)	1/10 (10.00)	1.00
**FAF**					
	Hyperautofluorescence		7/7 (100.00)	4/4 (100.00)	0.36
	Hypoautofluorescence		7/7 (100.00)	3/4 (75.00)	
**FA**					
	Hyperfluorescence		5/9 (55.56)	0/1	-
	Hypofluorescence		1/9 (11.11)	0/1	-
	Leakage		2/9 (22.22)	1/1	-
	No fill in lesions		2/9 (22.22)	0/1	-
	RPE atrophy		1/9 (11.11)	`0/1	-
	Scaring		1/9 (11.11)	1/1	-
**ICG**					
	Hypofluorescent focus		1/1	-	-
	Penetrating coiled vessel		1/1	-	-
	No leakage		1/1	-	-

N = number of eyes. In parenthesis—% of eyes in the group.

US- ultrasound; PVD- posterior vitreous detachment; OCT- optical coherence tomography; FAF- fundus autofluorescence; PED- pigment epithelium detachment; RPE- retinal pigment epithelium; ERM- epiretinal membrane; FA- fluorescein angiography. Mm = millimeter

Twenty-two eyes (22/32, 68.75%) presented with a pseudo mass on US, characterized as choroidal or sub retinal elevation of the peripheral retina (as opposed to choroidal thickening which does not cause an elevation). Internal reflectivity of the lesions, when available, was moderate to high in most eyes (23/26, 88.46%). On follow-up a concrete pseudo mass was found only in 4/11 (36.36%) eyes. When visible, the average base and thickness of the mass were 8.58±4.00 mm (n = 20, range 3.27–18.40, median 7.61) and 2.46±1.25 mm (n = 26, range 0.69–5.76, median 1.99) respectively at presentation, and 7.34±3.24 mm (n = 8, range 3.56–10.84, median 7.63) and 1.81±0.93 mm (n = 9, range 0.68–3.48, median 1.61) respectively on last US available during follow-up.

Prominent OCT findings at presentation included pigment epithelium detachment (PED) (6/20 eyes, 30.00%), macular RPE abnormalities (6/20 eyes, 30.00%), scarring (4/20 eyes, 20.00%) and subretinal fluid (6/20 eyes, 20.00%). With time the major OCT findings became mostly scarring (4/10 eyes, 40.00%) and subretinal deposits (3/10 eyes, 30.00%). Fundus autofluorescence (FAF) demonstrated simultaneously high and low autofluorescence of lesions in all eyes with available images at presentation (7/7, 100%), and in most eyes at follow-up (3/4, 75%).

FA findings were available in 9 eyes, with 5/9 (55.56%) exhibiting hyperfluorescence of the lesions, of which: 2/5 eyes (40.00%) with leakage, 2/5 (40.00%) with staining and 1/5 (20.00%) with pooling. The single patient who underwent ICGA testing demonstrated a hypofluorescent focus at the location of the lesion with gentle hyperfluorescence consistent with deep vasculature. A coiled vessel penetrating the lesion was observed during the early phase, with no leakage—this finding may represent an anastomosis.

No statistically significant difference in US measurements or OCT imaging findings was observed between the three disease subtypes (**Table 4**). Subgroup analysis of FA and ICGA findings was not feasible due to small sample size.

### Management

Treatment was conservative in 24/35 eyes (68.57%) with only timely follow-up examinations. The rest of the eyes (11/35, 31.43%) were treated with a various number of anti-VEGF (bevacizumab 1.25 mg/0.05 ml) injections, ranging from one to sixteen (mean 6.82±5.44, median 3.00). Indications for bevacizumab treatment were macular involvement in 1/11 eye (9.09%), lesions threatening the macula (4/11 eyes, 36.4%), vitreous hemorrhage (2/11 eyes, 18.18%), severely decreased vision in the fellow eye (LP and NLP respectively) in 2/11 eyes (18.18%), a very large lesion (US thickness 5.76mm with local retinal detachment) in 1/11 eye (9.09%), and a pre-existing diabetic macular edema in 1/11 eye (9.09%). Of note- two eyes with macular involvement were not assigned for treatment due to low visual potential of the involved eye. The clinical, sonographic and follow-up findings of patients in the conservative and anti-VEGF treatment groups are summarized in **Table 5**.

**Table 5:** Comparison of clinical and imaging characteristics, and prognosis of PEHCR patients treated with intravitreal bevacizumab to patients without treatment.

		Untreated	Treated	P
**At presentation:**				
Age		78.58 ± 9.22	79.91 ± 10.71	0.36
Male		9/24 (37.5)	6/11 (54.54)	0.56
VA average		0.82 ± 0.75	0.68 ± 0.62	0.31
Single Focus		20/24 (83.33)	7/11 (63.64)	0.23
Single Lesion		12/24 (50.00)	5/11 (45.45)	1.00
Clinical subtype:				
	Combined	8/24 (33.33)	2/11 (18.18)	0.45
	Exudative	10/24 (41.67)	2/11 (18.18)	0.26
	Hemorrhagic	6/24 (25.00)	7/11 (63.64)	0.06
US base (mm)		7.56 ± 3.51	10.47 ± 4.72	0.07
US thickness (mm)		2.16 ± 0.91	3.04 ± 1.69	0.01
**At follow-up:**				
Age		81.05 ± 9.21	81.29 ± 11.67	0.48
Male		6/19 (31.58)	4/9 (44.44)	0.81
Follow-up (months)		28.84 ± 27.62	32.89 ± 13.16	0.35
VA average		0.68 ± 0.55	0.99 ± 0.78	0.12
Single Focus		13/19 (68.42)	7/9 (77.78)	0.69
Single Lesion		11/19 (57.89)	6/9 (66.67)	0.70
US base (mm)		6.99 ± 3.52	8.40 ± 2.96	0.31
US thickness (mm)		1.75 ± 1.00	2.07 ± 0.87	0.35
Disease course:				
	Resolution	6/19 (31.58)	8/9 (88.89)	0.02
	Stable	9/19 (47.37)	0/9	0.03
	Progression to resolution	2/19 (10.53)	0/9	0.55
	Progression	2/19 (10.53)	1/9 (11.11)	1.00

N = number of eyes. In parenthesis—% of eyes in the group. Calculated values represent the average and standard deviation.

PEHCR- Peripheral Exudative Hemorrhagic Chorioretinopathy; VA- visual acuity in logMAR; US- ultrasound. Mm = millimeter

In the 9/11 (81.82%) eyes in the treatment group follow-up data of 32.89±13.16 months (range 6–58, median 35) was available, with a mean of 7.67±5.68 bevacizumab injections (range 1–16, median 6). Clinical disease resolution was observed in 8/9 eyes (88.89%), with only one eye (11.11%) continuing to deteriorate (P = 0.015). US thickness and the number of disease foci and of lesions within the foci decreased following the treatment yet the decrease was not statistically significant (**Table 6**). The decrease could not be validated statistically due to small sample size.

**Table 6:** Clinical characteristics of patients in the bevacizumab treatment group- prior and after treatment.

	Before treatment	After treatment	P value
VA	0.50 ± 0.26	0.99 ± 0.78	0.04961
BCVA	0.45 ± 0.28	0.96 ± 0.81	0.04963
Single focus	5/9 (55.56)	7/9 (77.78)	0.21
Single lesion	4/9 (44.44)	6/9 (66.67)	0.81
US base (mm)	8.19 ± 2.85	8.40 ± 2.96	0.13
US thickness (mm)	2.64 ± 1.67	2.07 ± 0.87	0.09

N = number of eyes. In parenthesis—% of eyes in the group. Calculated values represent the average and standard deviation.

VA- visual acuity in logMAR; BCVA- best corrected visual acuity in logMAR; US- ultrasound. Mm- millimeter.

**Fig 3:**
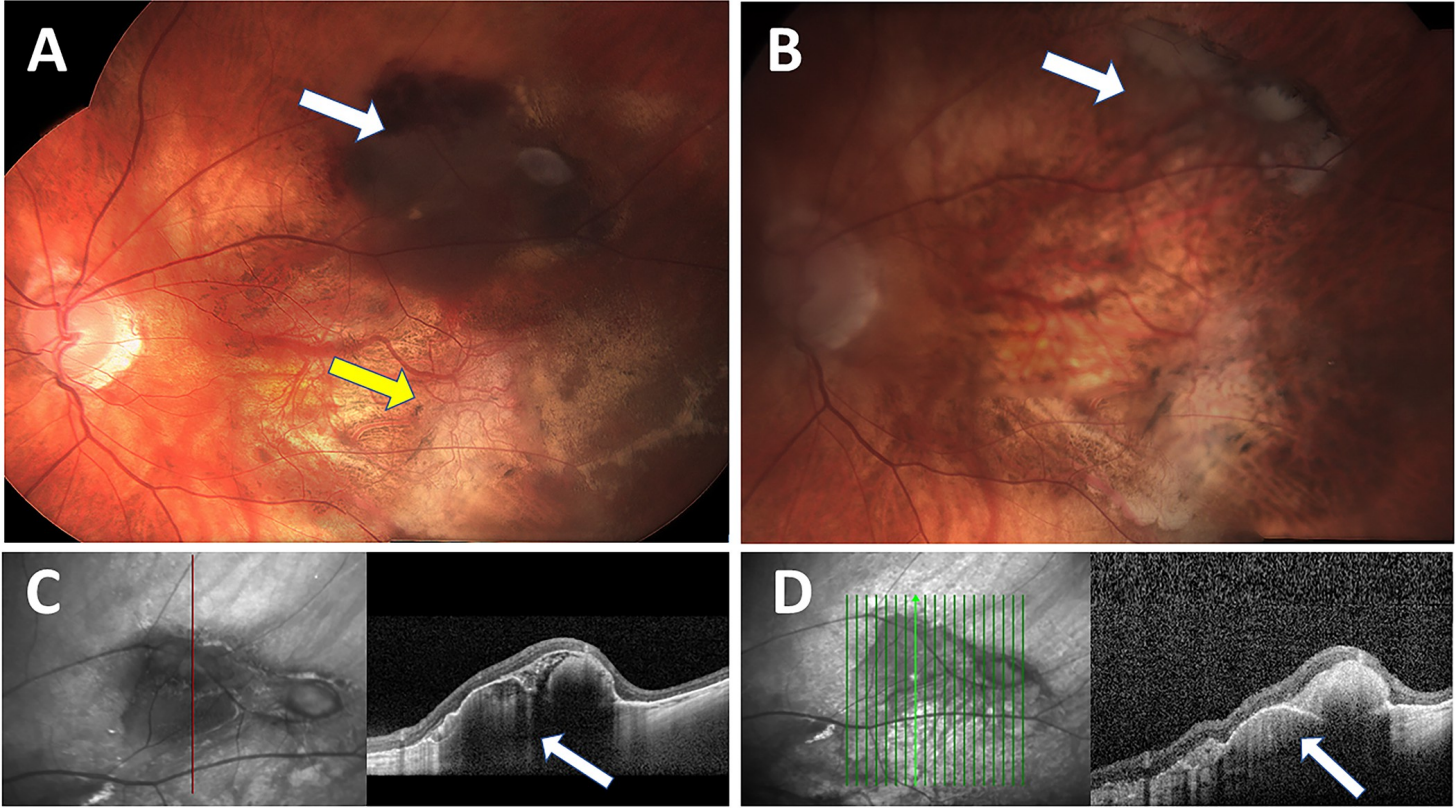
Clinical appearance and OCT findings before and after anti-VEGF treatment in a patient with macula threatening PEHCR. Color fundus photography of the same eye **A** before and **B** after 10 anti-VEGF intravitreal injections. **A** demonstrating a large elevated hemorrhagic lesion at the superior arcade consistent with hemorrhagic PEHCR (white arrow) old fibrotic lesion (yellow arrow), **B** resolution of the bleeding, clinical shrinkage of the lesion and residual atrophic changes (white arrow). **C+D,** OCT of the same eye at the level of the aforementioned lesion before and after treatment: **C** demonstrating a large hemorrhagic PED (white arrow) with no subretinal fluid. **D** After treatment the PED shrunk in size with a residual subretinal hyper-reflective layer consistent with scaring. No subretinal fluid is seen.

There was no statistically significant difference in the VA at last follow-up between the treated and untreated groups. **Fig 3** illustrates clinical and imaging findings of an eye before and after anti-VEGF treatment was applied.

### Disease course

Median follow-up time was 18 months (range 0–102). Changes in the subtype of disease over time were observed: 3/9 eyes (33.33%) with combined type at presentation transforming to exudative only, and 1/9 eyes (11.11%) transforming to hemorrhagic. Overall, 3/9 eyes (33.33%) of exudative and 2/10 eyes (20.00%) of hemorrhagic subtypes transformed into combined disease.

Changes in imaging findings over time were less pronounced than the clinical ones, but there was a trend toward more scarring and reduction in the number and size (both base and thickness) of lesions with time.

Clinical progression of disease was demonstrated in 3 eyes (two of which belonged to the same patient), whereas the rest of the eyes remained stable or improved over time. A female patient with bilateral involvement mentioned above (cases 11 and 12) presented to our service with a fulminant extensive disease involving the macula. At presentation VA in her left eye was NLP, and 0.22 logMAR in her right eye, which deteriorated to 1.6 logMAR over time. Disease phenotype was exudative in right and combined in the left eye. Intravitreal Bevacizumb treatment was administered to the right eye, but unfortunately progression was not halted. The third progressive case was a 72-year-old female with unilateral involvement (case no 5), with VA deterioration from 0.5 to 0.7 logMAR over a 24-month follow-up. Ophthalmic comorbidities included macular hole and atrophic optic disc, which may also account for her gradual visual deterioration. No treatment was initiated for this patient since PEHCR extent and location did not seem to justify such intervention.

Due to small sample size- most statistical analyses on the different subgroups were difficult to assess, but VA at follow-up was significantly worse in the combined subtype compared to hemorrhagic (P = 0.032). There was also more progression on slit lamp examination in the combined versus hemorrhagic group (22% versus 0%), but this was not statistically significant.

## Discussion

PEHCR is a rare clinically variable disorder, and as such-there is still much to learn about it. In our study we describe the clinical and imaging appearance of 35 eyes with PEHCR at diagnosis and at follow-up.

Demographic characteristics and past medical history of our cohort resembled previous descriptions of PEHCR [9, 10, 14]. An interesting finding was the relatively high prevalence of past malignancy in our cohort (10/32 patients, 31.25%), which has not been mentioned in previous reports of PEHCR. This fact, in combination with the referral diagnosis of malignancy, (malignant melanoma, lymphoma, metastasis, paraneoplastic syndrome etc.) [10–12]- emphasizes the challenge in the correct diagnosis of PEHCR.

Clinical variability at presentation is another factor making unequivocal diagnosis difficult. Our patients exhibited three distinct subtypes at presentation, sometimes transforming from one subtype to another (**Table 3**). The most severe clinical course seems to be related to the combined subtype with a significantly worse VA and a tendency towards worsening clinical appearance on follow-up, whereas the hemorrhagic subtype appears to be the most favorable. This observation is supported by previous studies [8]. Bilateral disease was found in only 9.38% of patients in our cohort, which is a much lower frequency than in previously reported (18.5–37%) [5, 8, 10, 18]. This difference implies that there may be environmental or ethnic disease modifying factors yet to be discovered. No particular anamnestic, clinical or imaging findings distinguished cases of bilateral involvement from unilateral ones in our cohort.

Several treatment modalities for PEHCR have been suggested previously, including cryotherapy, laser photoablation and anti-VEGF injections, with no statistically significant impact of either method thus far [8, 10, 15]. Previous reports have suggested intravitreal anti-VEGF injections as a treatment method to halt PEHCR progression and induce remission, especially in cases involving the macula or extending over a large area [7, 9, 14–18]. Except for one [18], all these reports were limited by short follow-up periods and small sample size (1 to 12 eyes), but demonstrated a favorable response to 2–3 injections, with resolution of subretinal fluid, hemorrhages, and some improvement in VA (although not statistically significant), sometimes requiring an additional injection if recurrence was observed. A recent study described 30 eyes with PEHCR treated with various anti-VEGF injections (11 Ranibizumab, 4 Bevacizumab and 3 Aflibercept) with a mean number of 7.7 injections per eye over a follow-up period of 30.7 months [15]. The outcome measure was VA improvement only- and was not meet. Zicarelli F et al. demonstrated reduction of macula-involving disease rate in the anti-VEGF treatment group [18]. In our cohort, repeated bevacizumab injections induced statistically significant resolution of the disease on clinical examination (P = 0.015). Additional parameters including lesion US thickness, the number of disease foci and number of lesions also showed a tendency toward reduction. There was no statistically significant difference in VA at last follow-up between the treated and untreated groups, which is an encouraging finding given the fact that the treated group had a more aggressive disease to begin with. In addition, VA of our cohort was affected mostly by other coexisting conditions, rendering it less relevant for disease monitoring.

Overall progressive disease occurred in 10.71% of eyes, whereas the rest remained stable or improved. Multimodal imaging findings also demonstrated a trend toward reduction in the amount and size of lesions. Shields et al. demonstrated a higher percentage of disease regression (89%), that may be explained by the shorter follow-up time [10]. Nevertheless, based on the literature thus far, it seems that PEHCR may be considered as a disease with a generally good prognosis for most patients.

The main limitation of our study, as in many rare diseases, is a relatively small sample size, overpassed only by a few other studies [5, 10, 15]. Follow-up time is another limitation, possibly due to mobilization limitations and concurrent medical conditions which are common in the elderly age group. This variability in follow-up time (ranging from 0 to 102 months) might affect the observed clinical course of disease. Additionally, since the concept of multimodal imaging became popular only recently, not all of our patients were tested with all the imaging modalities available. Specifically, since ICG wasn’t performed for most of the patients, PCV was ruled out based on clinical appearance only, which may be misleading in some cases. Further studies with more patients and longer follow-up periods are needed in order to better characterize this rare condition.

## Conclusions

PEHCR is a rare condition with high clinical variability. This is the first study to show statistically significant higher rate of disease resolution after intravitreal anti-VEGF injections.

## Supporting information

S1 Data (XLSX)
